# Global or local construction materials for post-disaster reconstruction? Sustainability assessment of 20 post-disaster shelter designs

**DOI:** 10.1016/j.dib.2015.05.027

**Published:** 2015-06-17

**Authors:** E. Zea Escamilla, G. Habert

**Affiliations:** Institute of Construction and Infrastructure Management, Chair of Sustainable Construction, Federal Institute of Technology (ETHZ), 8093 Zürich, Switzerland

**Keywords:** Sustainability, LCA, Transitional shelter, Construction

## Abstract

This data article presents the life cycle inventories of 20 transitional shelter solutions. The data was gathered from the reports 8 shelter designs [1]; 10 post-disaster shelter designs [2]; the environmental impact of brick production outside of Europe [3]; and the optimization of bamboo-based post-disaster housing units for tropical and subtropical regions using LCA methodologies [4]. These reports include bill of quantities, plans, performance analysis, and lifespan of the studied shelters. The data from these reports was used to develop the Life Cycle Inventories (LCI). All the amounts were converted from their original units (length, volume and amount) into mass (kg) units and the transport distance into ton×km. These LCIs represent the production phases of each shelter and the transportation distances for the construction materials. Two types of distances were included, local (road) and international (freight ship), which were estimated based on the area of the country of study. Furthermore, the digital visualization of the shelters is presented for each of the 20 designs. Moreover, this data article presents a summary of the results for the categories Environment, Cost and Risk and the contribution to the environmental impact from the different building components of each shelter. These results are related to the article “Global or local construction materials for post-disaster reconstruction? Sustainability assessment of 20 post-disaster shelter designs”[5]

## 1 Specifications table

**Table:** 

Subject area	*Sustainability*
More specific subject area	*Life cycle assessment, sustainable construction*
Type of data	*Tables*
How data was acquired	*Literature review*
Data format	*analysed*
Experimental factors	*None*
Experimental features	*None*
Data source location	*Worldwide*
Data accessibility	*The data is available at http://www.ifrc.org/PageFiles/95186/900300-Transitional%20Shelters-Eight%20designs-EN-LR.pdf*

## 2 Value of the data

• Describe the material demand (life cycle inventories) of several transitional shelters.
• The data comes from experiences on the field.
• Describes the cost and technical performance of transitional shelter, which is needed for their assessment.

## 3 Data, materials and methods

Three types of data are presented in this data article and are available in Supplementary files. First the lifecycle inventories for each shelter, this data represents the amount of construction material need to construct each shelter. Moreover this data present the transport distance that each amount of material was transported form its production site to the construction site. Second, Assessment results: this data presents the performance of each shelter on the proposed assessment categories Environment, Cost, and Risk and are associated to the article “Global or local construction materials for post-disaster reconstruction? Sustainability assessment of 20 post-disaster shelter designs” [5]. Finally, the contribution to environmental impacts. This data represent the contribution that each building component, foundation, structure, walls, roof and transport of construction material produces on the overall environmental impact and it is related to the article article “Global or local construction materials for post-disaster reconstruction? Sustainability assessment of 20 post-disaster shelter designs” [5]. Finally, a digital representation of the shelters is provided.

### 3.1 B1 Afghanistan bamboo

This shelter was built to act as a shell to protect occupants living in tents. Each shelter contains one tent, erected inside the structure. It is rectangular in plan and has 1.8 m tall side walls and a gable roof. The covered floor area is approximately 9 m×4.3 m. The frames are constructed from bamboo poles. The frames are connected using plywood gusset plates and bolts. The walls and roof are plastic sheeting, and are supported on the bamboo frame and purlins. The floor is compacted soil. The shelter frames were shop fabricated in the camp and transported to the construction site. The frames are embedded into the ground for support [2].

### 3.2 B5 Indonesia bamboo

The rectangular bamboo frame structure measures 6 m×4 m on plan and has a hipped roof of terracotta tiles laid on bamboo matting and laths. The frame has woven bamboo matting walls, a door at the front and two windows on each side. The back section has a raised floor which forms a sleeping area constructed from bamboo joists and panelling. The floor void has been filled with rubble confined by a low masonry wall all round. The structure is braced with bamboo members on all sides which provides stability with an additional roof truss in the centre. The shelter is supported by five bucket foundations with a length of bamboo cast in to connect to the four main columns. The frame connections are pinned using bamboo pegs and then secured with rope. The roofing and flooring are fixed with nails [1].

### 3.3 B8 Philippines bamboo

This shelter is a rectangular structure with 2 or 4 slopes on the roof depending on the configuration. The inner areas is approximately 3 m×6 m. The roof extends 1 m on each direction to provide protection from the rain. The exterior is composed of bamboo based frames using bamboos with diameters between 8 and 10 cms. The frames are cladded with flattened bamboo and chicken mesh. The cladding can be applied outside and inside or only outside depending on the external hazards. The cladding layer is covered with a mortar cement plaster with thickness from 1 cm to 2 cms. The frames are supported by a line of concrete hollow blocks. The roof consists of a bamboo poles structure and galvanized steel sheets. The joints between bamboo elements are reinforced with steel elements and slurry concrete. The design can be customized with several options for doors and windows depending on the desired configuration [4,6–8].

### 3.4 C2 Bangladesh concrete / timber

This shelter is has reinforced concrete columns, a steel framed hip roof with metal roofing and bamboo mat walls. The total covered area is approximately 4.5 m×3.2 m, and there is one door and three windows.

The floor is raised above existing grade, and a short brick wall is provided around the perimeter to resist flood waters and windblown rain. The 8 concrete columns are embedded approximately 1.5 m into the ground. The roof truss is constructed with steel angles and is anchored to the concrete columns. The foundation consists of the 8 embedded columns, and a perimeter concrete grade beam. There are wooden beams between the columns approximately 2.1 m above the first floor, which allow the addition of a mezzanine level to the shelter. The shelter is designed to be easily moved by unbolting the columns and roof frame with hand tools and the materials can be re-used as a part of permanent housing reconstruction. Additionally it is designed so that a mezzanine level can be built to provide storage space in case of floods [2].

### 3.5 C6 Pakistan brick

This shelter is a rectangular structure with a flat roof with approximate dimensions of 4.8 m×3.9 m. Walls are built with 230 mm thick unreinforced fire burned brick walls supporting the roof. The roof is constructed with ceramic tiles supported on steel beams, and a cement plaster coating is placed on top of the tiles. The foundation consists of unreinforced brick footings and foundation walls. The mud plastered floor is raised a minimum of 610 mm above the surrounding ground surface. As designed, the shelter has one door and one window, along with air vents near the top of the walls [2].

### 3.6 C8 Philippines concrete

This shelter is a rectangular structure with a single pitch roof and a covered floor area of approximately 4.8 m×3.7 m. The shelter is supported on concrete piers and footings such that the first floor is raised approximately 750 mm above grade. The floor and roof are framed with coconut wood beams and joists. The floor is plywood and the roof is corrugated metal roofing. The exterior walls consist of amakan (woven panels of bamboo or palm leaves) fastened to the coconut wood frame. The light weight wood frame can be lifted off the concrete piers and moved to a different location by a small number of people. As designed, the shelter has one door and two windows [2].

### 3.7 C9 Sri Lanka concrete / timber

This shelter is a rectangular structure with a gable roof and an enclosed floor area of approximately 3.5 m×2.8 m with an additional covered veranda of approximately 3.5 m×2.8 m. The exterior walls are built with unreinforced bricks with six reinforced masonry piers. All masonry blocks are fabricated by the shelter occupants prior to construction. The roof consists of coconut wood rafters and purlins supporting corrugated iron sheet roofing. The compacted earth and concrete floor is raised above the surrounding ground surface. The perimeter walls extend into the ground, and are supported on brick footings. The modular construction for the shelter allows for expansion in both horizontal directions with only minor modifications to the core shelter. As designed, the shelter has one door and one window [2].

### 3.8 C11 Nicaragua ferrocement

This shelter is a rectangular structure with a gable roof and a covered area of approximately 3 m×6 m with additional division walls. The exterior is built with ferrocement panels of 0.5 m×2.5 m. All the panels are prefeabricated either at small facory or locally depending on the availiability. The reinforcement consit on a lower and upper concrete ring. The roof consist of a galvanized steel sheets. The floor is a slat of poor concrete. The constructive system used on this shelter allows for its expansion both vertically and horizontally. The shelter can be customized with several options for doors and windows depending on the desired configuration [3].

### 3.9 S4 Haiti steel

The shelter consists of a galvanised rectangular steel frame with an 8.5° mono-pitch roof and a suspended floor. The height to the eaves is 2.55 m and 3 m to the ridge and there is no bracing. The shelter is 3×6 m^2^ on plan and has 6 columns spaced on a 3 m grid, fixed to 800×800×400 mm^3^ rectangular reinforced concrete foundations using a 300×300×6 mm^3^ base plate and four ordinary bolts per base. The raised floor is also supported by 13 additional stub columns on 100×100×6 mm^3^ base plates bearing directly on to the soil. The main structure is three primary frames with rectangular hollow section columns. The roof cladding is corrugated steel sheeting nailed to steel secondary roof members spaced at 0.75 m intervals spanning between the three primary frames. Timber studs are screwed to the steel members and the plastic wall sheeting is attached to this. Additional timber sub-framing is used to form windows and doors [1].

### 3.10 S5 Indonesia steel

The structure consists of a cold rolled, hot dip galvanised steel frame with a pitched roof of 24.3° and a raised floor. The height is 2.8 m to the eaves and 4.15 m to the ridge. The platform area of the shelter is 25 m^2^ with a cantilevering balcony at opposite sides front and back and a cantilevering roof covering the balconies. There are 6 columns fixed using column base plates nailed directly into the ground. Metal roof sheets are screwed to steel purlins spanning between primary roof beams. Limited lateral stability is provided by timber plank wall cladding fixed to timber studs that are in turn screwed to the steel frame. The floor consists of timber planks spanning between steel joists [1].

### 3.11 S10 Vietnam steel

The shelter is a galvanised lightweight steel frame with plywood walls and a corrugated steel sheet roof. It has a covered area of 3.6×8.4 m^2^ on plan including a living area of 3.6×7.2 m^2^. The roof has a pitch of 16.5°. The height of the roof varies from 3.2 m at the eaves to 4.6 m at the ridge. There are two doors, one at the side and one at the front, and a cantilevered canopy projecting 1.3 m beyond the door to form a porch. There are 12 columns, six of which have screw in ground anchor foundations, connected in pairs by a braced truss to form a moment frame. The stability system is formed by these three moment frames tied together by two further moment frames on each edge of the building. There is steel tie bracing underneath the roof sheeting. The shelter has a 100 mm thick concrete slab base cast over the screw anchor foundations and floor tie beams. There is a low, non-structural, 0.5 m, brickwork wall providing a degree of flood protection [1].

### 3.12 W3 Burkina Faso timber

This shelter is a rectangular timber frame with a pitched roof and a covered floor area of 2.7 m×1.8 m. The frame has plastic sheeting for both roof and wall covering, and one door on each short side. The wall frame is made from timber panels that are pre-fabricated on the ground. The timber roof structure is nailed to these panels. Both walls and roof are reinforced with wire cross bracing. There is a knee braced timber framed along the roof ridge which supports the roof panels, and provides stability during construction. Wall and roof covering is fastened to the timbers using flat-head nails [2].

### 3.13 W4(A) Haiti timber

This shelter is a rectangular timber framed structure with a gable roof and a covered floor area of approximately 21 m^2^. Wall consists of wood studs with plywood sheathing, and the roof consists of metal roofing on wood purlins and trusses. The trusses are supported on wood posts within in the perimeter walls. The wood trusses can be pre-manufactured and shipped to the construction site. The foundation consists of concrete piers in the four corners and a stone masonry wall in-between the piers. The floor is a cast-in-place concrete slab. As designed, the shelter has only one door and one window [2].

### 3.14 W4(B) Haiti timber

This shelter is a rectangular timber framed structure with a gable roof and a covered floor area of approximately 3.6 m×4.9 m with a covered porch measuring approximately 3.6 m×1.8 m in front. The floor is constructed with wood joists, and the walls are constructed with wood studs. Both are supported by built-up timber posts. The roof is framed with wood trusses that can be pre-manufactured and shipped to the site. The roof extends over the porch to provide cover. Floors and walls are covered with plywood, and the roof is covered with metal panels. The bottom of the built-up timber posts are encased in concrete and embedded in the ground. The design includes one door in the front and back walls, and louvred wall openings [2].

### 3.15 W4(C) Haiti timber

This shelter is a rectangular timber framed structure with a gable roof and a covered floor area of approximately 5.4 m×3.7 m with a covered porch measuring approximately 1.8 m×3.7 m. The roof has wood and corrugated bituminous roofing supported on timber purlins and trusses. The exterior walls are wood framed, and the wall infill is constructed using a traditional technique called clissage, which consists of thin slats of wood woven between the wall framing. The foundation consists of wood posts embedded in concrete piers, and the floor is an elevated concrete slab supported by a short masonry wall between the wood posts. As designed, the shelter has one door and two windows. The shelters were designed to be accessible by persons with reduced mobility and individual modifications were made according to personal needs [2].

### 3.16 W5 Indonesia timber

The shelter is a timber framed structure with palm roofing and walls. It measures 4.5 m×4 m on plan and is 3.35 m tall to the ridge beam and 2.4 m to the eaves. It has a pitched roof of 23.6°. There is no bracing, but some stability is provided by three portal frames tied together by horizontal members at ground, eaves and ridge level. Each portal frame is made up of two or three columns and a roof truss with rafters and corner bracing members. The corner bracing in the frames provides lateral stiffness. Secondary non-structural members include: floor joists, roof joists spanning between rafters and transoms to support palm matting wall panels. The shelter has a suspended floor. This is assumed to be coconut wood boarding spanning between the floor joists. The columns are embedded into concrete bucket foundations that sit directly on the ground [1].

### 3.17 W6 Pakistan timber

The shelter consists of 7 triangular frames, connected by a ridge pole. The ridge pole is supported by two 2.74 m high vertical columns at each end. The shelter is 4.3 m×5.7 m on plan. It has a low (0.9 m) brick wall constructed inside the frame to provide protection against flood damage and retain warmth. The roof is pitched at 44° and is made of corrugated steel sheeting. The sheeting is nailed to purlins that span between the frames. The roof sheeting is laid on top of locally available insulating material and plastic sheeting. The foundation of the shelter is provided by burying the rafters and columns approximately 0.3 m in to the ground on top of stone footings. Guy ropes over the roof sheeting have been used to help prevent uplift under wind loads [1].

### 3.18 W7(A) Peru timber

The shelter has a Bolaina (Bolayna) timber braced frame, measuring 3 m×6 m on plan with a single pitched roof at four degrees. The shelter is clad with tongue and groove solid timber board walls and a corrugated fibre cement sheet roof. It is 2.4 m high and stands on a new or existing concrete floor slab. In instances where a new slab has been used, wire ties wrapped around nails have been cast into the slab and attached to the frame at all column locations to resist uplift. Where existing slabs have been used the shelter has been staked to posts installed outside the slab. The shelter is constructed as 6 panels which are then nailed together using connecting wooden members, connecting plates and plastic strapping. A central roof edge beam is attached to the panels and are purlins nailed on top of this to support the roof [1].

### 3.19 W7(B) Peru timber

The structure is a rigid box consisting of braced frames in both directions. The braced frames provide lateral stability. The eucalyptus timber frame has a flat roof and is covered with stapled plastic sheeting and nailed palm matting on all faces. The shelter is 2 m high and 3 m×6 m on plan. The bracing consists of crossed twisted wires. The 75 mm diameter columns are connected horizontally with 50 mm diameter horizontal members. The foundation and floor consists of an unreinforced concrete slab with cast in wire ties. The connections between members are made using bent nails [1].

### 3.20 W8 Philippines timber

This shelter is a rectangular structure with a gable roof and a covered floor area of approximately 4.0 m×5.0 m with a covered bathroom and vestibule of approximately 4.0 m×1.5 m. The exterior walls have a half height concrete masonry wall with wood framing on top up to the eaves. The roof consists of timber trusses and purlins supporting corrugated metal roofing. The roof framing is supported by eight precast concrete columns located within the exterior walls. The concrete columns and masonry walls are embedded in the ground, and the plans do not specifically call for footings. The floor is a cast in place concrete slab, and the bathroom has a below grade septic tank. The modular construction for the shelter allows for expansion in both horizontal directions with only minor modifications to the core shelter. It is also possible to deconstruct the shelter for relocation and/or to be included in permanent construction. As designed, the shelter has two doors and two windows [2].

## 4 Methods

The methodology to produce the data here presented is described on the article “Global or local construction materials for post-disaster reconstruction? Sustainability assessment of 20 post-disaster shelter designs” [5].
